# Supplementary material for: Stability of Intracellular Protein Concentration under Extreme Osmotic Challenge
Source: Cells. 2021 Dec 14;10(12):3532. doi: 10.3390/cells10123532 (PMC8700764; doi:10.3390/cells10123532)
Supplement: Supplementary file 1 [file cells-10-03532-s001.zip › cells-1463969-supplementary.pdf]

Supplement

Hollembeak JE, Model MA

I. Amiloride-induced apoptosis

A sodium channel inhibitor amiloride caused dose-dependent cell death in 50% DMEM (Fig. S1)

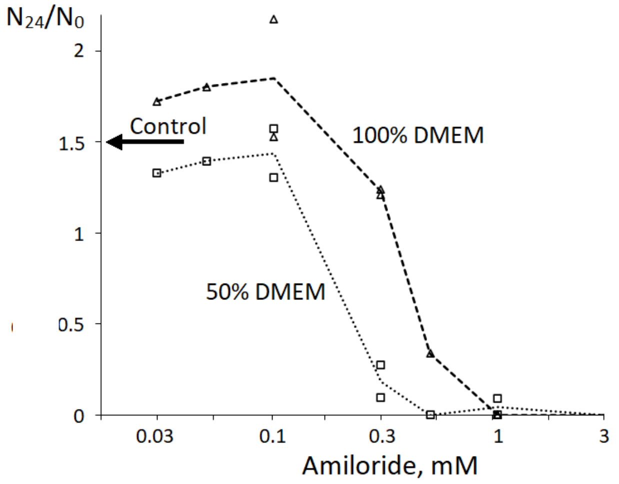

Figure S1. The effect of amiloride on HeLa in 100% DMEM and 50% DMEM. The proliferation factor N_{24}/N_0 was defined as the relative increase in the number of cells counted in the same area over 24 h; it was close to 1.5 in control samples without amiloride. All cells survived in 0.1 mM amiloride, and all died in 1 mM amiloride in both media; however, a large difference was observed when the drug was present at 0.3-0.5 mM.

Amiloride-induced cell death in 50% DMEM occurred rapidly, with many cells showing characteristic apoptotic features: blebbing, caspase staining, and loss of mitochondrial potential (Fig. S2). This pattern of apoptosis was very similar to that in response to actinomycin D [Kasim et al, 2013], only developing much faster; a significant proportion of cells became apoptotic after only 3 h of treatment.

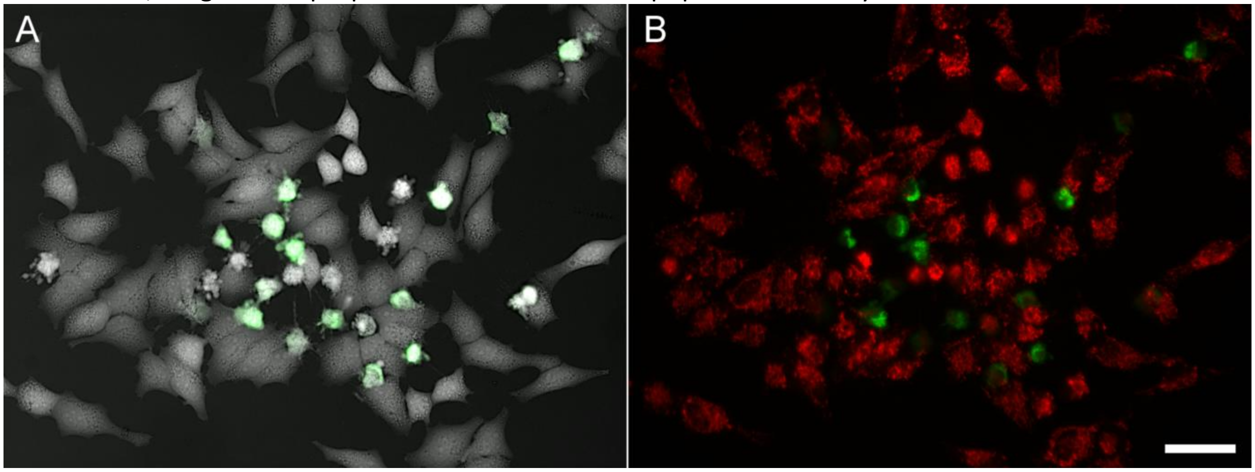

Figure S2. Amiloride-induced apoptosis after a 4-h incubation in 50% DMEM. (A) Overlay of TTD and NucView staining. (B) Overlay of NucView (green) and TMRE (red) staining. Scale bar, 50 µm.

II. Calibration of TIE

TIE computes the phase delay Δϕ from two or more mutually defocused bright-field images. The usefulness of this parameter comes from the fact that it is quantitatively related to object thickness and refractive index:

$$\Delta\phi = \frac{2\pi h}{\lambda_0}[n - n']$$

or, in terms of integral quantities,

$$A \cdot \underline{\Delta\phi} = \frac{2\pi V}{\lambda_0}[n - n']$$

The refractive index is mostly a function of protein concentrations in the cell (c) and the medium (c'):

$$n - n' \approx 0.185(c - c')$$

Unlike proteins, inorganic salts have only a minimal effect on refractive index. For example, Δn/Δc for NaCl or KCl is close to 0.01 1/M [Petrache et al, 2006; Tan and Huang, 2015], which translates into Δn = 0.003 for the typical salt concentration in mammalian cells. This can be compared to organic molecules with Δn/Δc ≈ 0.185 ml/g or Δn = 0.37 for 0.2 g/ml. That alone gives a hundred-fold difference, which is in fact much larger because salts are present both inside and outside the cell, whereas proteins are contained mostly inside. As for the small organic metabolites, they add to the refractive index roughly in proportion to their mass. If the contribution of small metabolites to osmolarity is 0.1M and their average molecular weight is 100, they may contribute 5% to the overall refractive index. (This number would be much larger in marine invertebrates, which are rich in organic osmolytes).

We use the TIE code written by Gorthi and Schonbrun [2012], which is based on two bright-field images BF1 and BF2. The images were collected with a 20x objective through a 485 nm bandpass filter at a nominal vertical separation 5 µm. To achieve uniform background, we crop images to square sections with sides usually not exceeding 500-600 pixels and with boundaries passing through empty areas of an image and equalize the average intensities of BF1 and BF2 prior to processing. The latter is done either manually or using a simple ImageJ plugin written by R. Clements (http://drosophila.biology.kent.edu/users/rclement/extras/equalizer.html).

The TIE output depends on the accurate knowledge of Δz. Unfortunately, the accuracy of the vertical travel of the stage (or of the objective) is not routinely tested and cannot be easily corrected. Therefore, we calibrate TIE directly in terms of protein concentration, bypassing the explicit determination of the phase. To do that, we assume that the result of TIE computation is not necessarily equal but only proportional to Δϕ:

$$c - c' = p\frac{T}{V}$$

where T is the output of TIE integrated over cell volume and p may differ from the theoretical $0.86\lambda_0$ = 0.417. The problem then becomes equivalent to finding the coefficient p.

To measure p experimentally without having a calibration object of defined size and shape, we perform measurements of T and V on the same cells in two AB9-containing buffers with different concentrations of protein (BSA) and use the fact that within a short time period, the total amount of intracellular protein remains unchanged. If V_1 and V_2 are the volumes of the same cell in two solutions with BSA concentrations c_1' and c_2' and T_1 and T_2 are the respective TIE values integrated over the same cell, we obtain three linear equations with three unknowns c_1, c_2, and p:

$$T_1 = \frac{1}{p} V_1 (c_1 - c_1')$$
$$T_2 = \frac{1}{p} V_2 (c_2 - c_2')$$
$$V_1 c_1 = V_2 c_2$$

where the latter equation represents the condition of dry mass conservation. We solve these equations for p:

$$p = \frac{V_2 c_2' - V_1 c_1'}{T_1 - T_2}$$

This result becomes particularly simple if the first measurement is performed in a protein-free buffer ($c_1' = 0$). Then p can be found from the slope of the line

$$\frac{T_1 - T_2}{V_2} = \frac{1}{p} c_2'$$

There is no need to ensure isosmolarity of the two solutions, which is especially convenient since the osmometer is not a standard piece of equipment in every lab.

The results are shown in Fig. S3. There is a large difference between the empirical slope 1.36 and the theoretical 1/0.417 = 2.4. We did not attempt to determine if that was due to systematic errors in the vertical travel or if other factors have also played a role. In earlier work, the slope was found to be 1.02 [Mudrak et al, 2018] and on some days, we observed significant deviations from the value 1.36. For this reason, only the relative changes in PC are reported in this work.

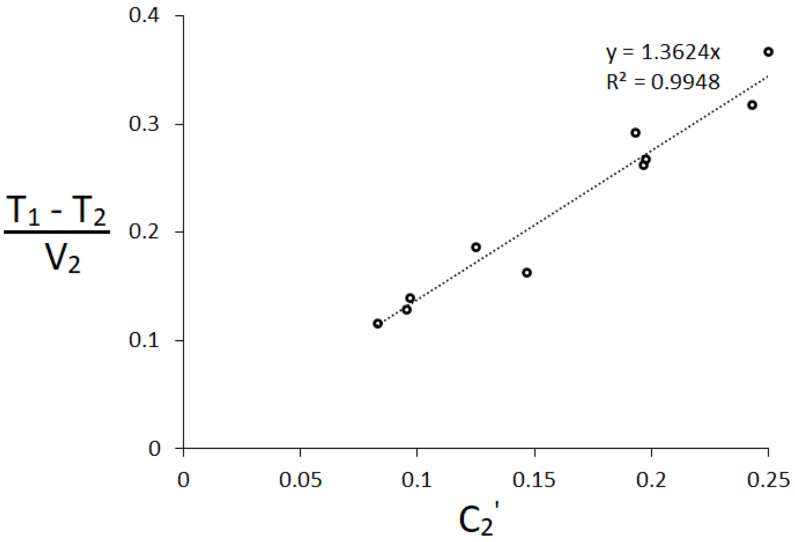

Figure S3. The results of TIE calibration; each point represents the averaged result of a separate experiment, in which at least several cells or groups of cells were analyzed. Reproduced from [Model, 2021] with permission.

References

Gorthi SS and Schonbrun E. (2012) Phase imaging flow cytometry using a focus-stack collecting microscope. Optics Letters, 37:707-709.

Kasim NR, Kuželová K, Holoubek A, Model MA. (2013) Live fluorescence and transmission-through-dye microscopic study of actinomycin D-induced apoptosis and apoptotic volume decrease. Apoptosis, 18:521-532.

Model MA. (2021) Studying cell volume beyond cell volume. In: Current Topics in Membranes; M.A. Model and I. Levitan, eds. Volume 88 (In press)

Mudrak NJ, Rana PS, Model MA. (2018) Calibrated brightfield-based imaging for measuring intracellular protein concentration. Cytometry A, 93:297-304.

Petrache HI, Zemb T, Belloni L, Parsegian VA. (2006) Salt screening and specific ion adsorption determine neutral-lipid membrane interactions. Proceedings of the National Academy of Sciences, 103:7982-7987.

Tan CY, Huang YX. (2015) Dependence of refractive index on concentration and temperature in electrolyte solution, polar solution, nonpolar solution, and protein solution. Journal of Chemical and Engineering Data, 60:2827-2833.
